# Retinal Tissue Shows Glial Changes in a Dravet Syndrome Knock-in Mouse Model

**DOI:** 10.3390/ijms24032727

**Published:** 2023-02-01

**Authors:** Juan J. Salazar, Andrea Satriano, José A. Matamoros, José A. Fernández-Albarral, Elena Salobrar-García, Inés López-Cuenca, Rosa de Hoz, Lidia Sánchez-Puebla, José M. Ramírez, Cristina Alonso, Valentina Satta, Inés Hernández-Fisac, Onintza Sagredo, Ana I. Ramírez

**Affiliations:** 1 Instituto de Investigaciones Oftalmológicas Ramón Castroviejo, Grupo UCM 920105, IdISSC, Universidad Complutense de Madrid, 28040 Madrid, Spain; 2 Facultad de Óptica y Optometría, Departamento de Inmunología, Oftalmología y ORL, Universidad Complutense de Madrid, 28037 Madrid, Spain; 3 Preclinical and Translational Pharmacology, Department of Pharmacy, Health and Nutritional Sciences, University of Calabria, 87036 Rende, Italy; 4 Facultad de Medicina, Departamento de Inmunología, Oftalmología y ORL, Universidad Complutense de Madrid, 28040 Madrid, Spain; 5 Centro de Investigación Biomédica en Red en Enfermedades Neurodegenerativas (CIBERNED), Instituto de Salud Carlos III, 28031 Madrid, Spain; 6 Instituto Universitario de Investigación en Neuroquímica, Departamento de Bioquímica y Biología Molecular, Facultad de Medicina, Universidad Complutense, 28040 Madrid, Spain; 7 Instituto Ramón y Cajal de Investigación Sanitaria (IRYCIS), 28034 Madrid, Spain

**Keywords:** Dravet syndrome, retina, astrocytes, microglia, retinal ganglion cells, GABAergic amacrine cells, Syn-Cre/Scn1a^WT/A1783V^ mice

## Abstract

Dravet syndrome (DS) is an epileptic encephalopathy caused by mutations in the *Scn1a* gene encoding the α1 subunit of the Nav1.1 sodium channel, which is associated with recurrent and generalized seizures, even leading to death. In experimental models of DS, histological alterations have been found in the brain; however, the retina is a projection of the brain and there are no studies that analyze the possible histological changes that may occur in the disease. This study analyzes the retinal histological changes in glial cells (microglia and astrocytes), retinal ganglion cells (RGCs) and GABAergic amacrine cells in an experimental model of DS (Syn-Cre/Scn1a^WT/A1783V^) compared to a control group at postnatal day (PND) 25. Retinal whole-mounts were labeled with anti-GFAP, anti-Iba-1, anti-Brn3a and anti-GAD65/67. Signs of microglial and astroglial activation, and the number of Brn3a+ and GAD65+67+ cells were quantified. We found retinal activation of astroglial and microglial cells but not death of RGCs and GABAergic amacrine cells. These changes are similar to those found at the level of the hippocampus in the same experimental model in PND25, indicating a relationship between brain and retinal changes in DS. This suggests that the retina could serve as a possible biomarker in DS.

## 1. Introduction

Dravet syndrome (DS), also called severe myoclonic epilepsy of infancy, is a rare genetic form of severe drug-resistant epilepsy, affecting 1:20,000 subjects. The onset of the disease begins at very early developmental age in previously healthy children, with a first generalized seizure at 5–8 months of age. The seizures become prolonged and frequent and highly resistant to antiepileptic drugs [1], which is related to cognitive impairment, developmental delays, hyperactivity, dysautonomia, attention deficit, language impairment, autistic features, and a high rate of sudden death (10–20%) [2,3,4,5] observed in these patients.

Approximately 70–80% of these patients have a haploinsufficiency in the *Snc1a* gene encoding the alpha subunit of a voltage-dependent sodium channel type 1 (Nav1.1) leading to a loss of function in this channel [6,7]. GABAergic interneurons are particularly affected [8], where the Nav1.1 channel is mainly located and the consequent imbalance between excitation and inhibition triggers brain hyperexcitability and seizures [9], altering the correct functioning of neural networks involved in cognitive processes [10].

Given the genetic origin of DS, it has been possible to develop transgenic, knock-out and knock-in experimental models that are enabling to discern the pathophysiological mechanisms (cellular and molecular) related to this pathology, which may greatly facilitate the development of new therapies for this disease [9,11,12,13,14,15,16,17,18,19,20,21]. In this study we used a heterozygous conditional knock-in mouse model carrying a nonsense mutation (A1783V) in the *Scn1a* gene that is expressed exclusively in CNS neurons (Syn-Cre/Scn1a^WT/A1783V^) [22].

Given Dravet syndrome is a neurodevelopmental disease, the phenotype of the animal model has been studied at a post-weaning stage, since preclinical evaluation at this stage of development can provide valuable information for a precision medicine approach aimed at these patients. Characterization of this model has shown that the A1783V mutation expressed in the nervous system is associated with spontaneous seizures and cognitive-behavioral alterations reminiscent of those observed in patients with DS [22]

Glial reactivity and neuroinflammatory processes have been shown to be present in childhood epileptic processes [23,24,25] and therefore cytokines may exert a neuromodulatory role directly impacting neuronal excitability [26]. Indeed, astrocytes have been documented to be involved in the pathogenesis of epilepsy [27], regulating neurotransmitter and ion concentrations [28,29].

In our experimental model of DS (Syn-Cre/Scn1a^WT/A1783V^), the behavioral alterations become more evident at 25 days of life with inflammatory events in the prefrontal cortex and dentate gyrus of the hippocampus expressed as an increase in GFAP (astrocyte marker) and Iba-1 (microglia marker) immunoreactivity, in addition to morphological changes compatible with activated astrocytes and microglia [22]. As observed by Martín-Suárez et al., in our model neural stem cells also acquire a reactive phenotype with increased mitotic activity, which might be contributing to reactive gliogenesis [1].

In several pathologies affecting the central nervous system, such as neurodegenerative diseases, correlation between changes occurring in the brain and those occurring in the retina has been shown. This has been observed at functional and structural level in patients and has been corroborated in experimental studies in different animal models of various neurodegenerative pathologies such as Alzheimer’s disease [30,31,32,33,34] or amyotrophic lateral sclerosis (ALS) [35,36] (ALS). The retina or neural portion of the eye is actually part of the central nervous system and due to its greater accessibility, it can be considered as a “window” that allows us to determine the changes that occur at the cerebral level in the event of damage. The alterations undergone by the cells located in different layers of the retina could be good predictive and prognostic biomarkers for future treatment. DS is a neurodevelopmental disease and in our experimental model presents a particularly altered motor and cognitive phenotype at 25 days of life, therefore, preclinical evaluation at this developmental stage may provide valuable information for a precision medicine approach aimed these patients. We have changed the sentence to make it grammatically clearer.

To our knowledge, there are no studies that analyze the changes occurring at the histological level, either in human retinas or in experimental models with DS. Therefore, the present work aims to analyze the possible changes that may occur in in glial cells (astrocytes and microglia), in an experimental model of DS (Syn-Cre/Scn1a^WT/A1783V^), in order to establish a correlation with the alterations observed in some specific cerebral areas of these animals.

Prior to diagnostic confirmation, visual alterations (visual field and ocular motility) have been observed in DS patients [37,38] and therefore, as a preliminary study, we examined the possible changes that may occur in GABAergic amacrine cells and glutamatergic ganglion cells of the retina in our animal model of DS.

## 2. Results

### 2.1. Morphology and Distribution and Quantification of Retinal Microglia

Microglial cells from wild type (WT) and Dravet syndrome (DS) animals were distributed, forming plexuses in the outer plexiform layer (OPL), inner plexiform layer (IPL), and nerve fiber layer-ganglion cell layer (NFL-GCL).

#### 2.1.1. Outer Plexiform Layer (OPL)

**Figure 1:**
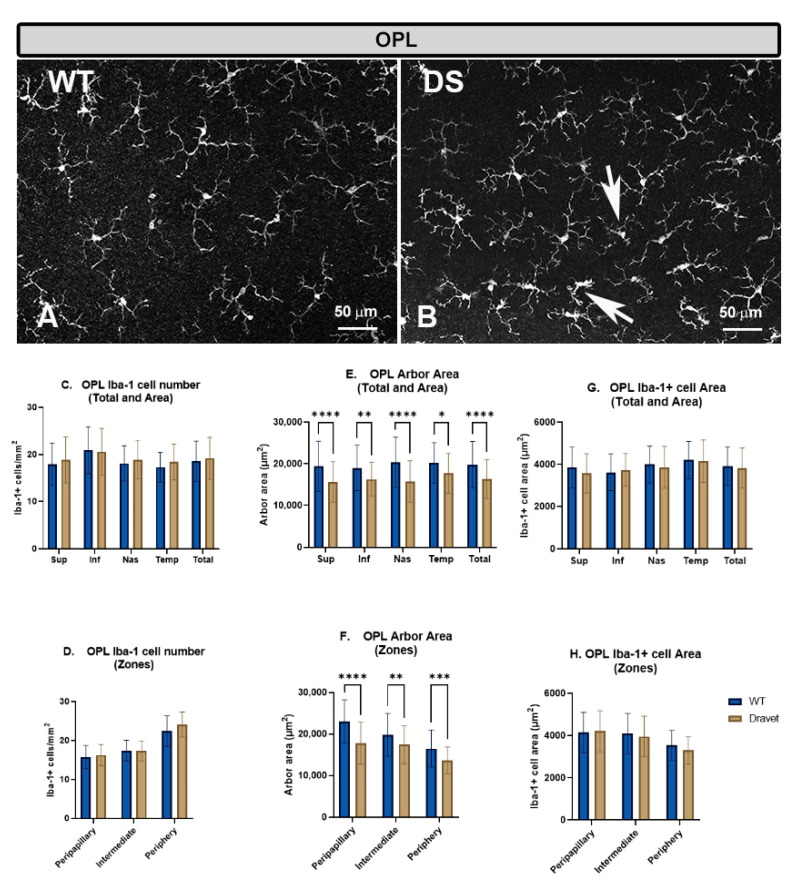
Microglial cells in the outer plexiform layer (OPL). (**A**,**B**): retinal whole-mounts labeled with anti-Iba-1 at 20× magnification. Arrows indicate Iba-1+ microglial cells with retraction of the processes in the Dravet syndrome (DS) animals. (**C**,**D**): number of Iba-1+ microglial cells per retinal area 0.1502 mm^2^ by total retina and area € and by zone (**D**). (**E**,**F**): Arbor area of Iba-1+ microglia by total retina and area (**E**) and by zone (**F**). (**G**,**H**): Iba-1+ cell area by total retina and area (**G**) and by zone (**H**). Two-way ANOVA. Data expressed as mean value (±SD). * *p* < 0.05, ** *p* < 0.01, *** *p* < 0.001, **** *p* < 0.0001. Number of retinas used in the experiment: WT animals n = 6, DS animals n = 6. Sup: superior; Inf: inferior; Nas: nasal; Temp: temporal.

In the OPL, the microglia of the WT and DS animals were arranged parallel to the retinal surface, forming a plexus. The cells had a branched appearance with small somas, from which emerged primary processes that divided into secondary and tertiary ones (Figure 1A,B).

The **number of microglia** in the OPL showed no significant changes in the DS compared to the WT animals, both in the total number of cells per retina (Figure 1C) and when the analysis was performed by areas (superior, inferior, nasal and temporal) (Figure 1C) or by zones (peripapillary, intermediate and peripheral) (Figure 1D).

However, the quantification of the **microglial arbor area** in this layer showed a significant decrease in the DS compared to the WT animals, indicating a retraction of microglial processes, both when the analysis was in the total retina (*p* < 0.0001) (Figure 1E) and when the analysis was performed by areas (superior: *p* < 0.0001, inferior: *p* < 0.01, nasal: *p* < 0.0001 and temporal *p* < 0.05) (Figure 1E) or by zones (peripapillary: *p* < 0.0001, intermediate: *p* < 0.01 and peripheral: *p* < 0.001) (Figure 1F). This retraction of the processes is a morphological sign indicative of microglial activation.

The quantification of the **area occupied by each microglial cell** in the OPL showed no significant changes in the DS compared to the WT animals, both in the total number of cells per retina (Figure 1G) and in the analysis by areas (superior, inferior, nasal and temporal) (Figure 1G) or by zones (peripapillary, intermediate and peripheral) (Figure 1H).

#### 2.1.2. Inner Plexiform Layer (IPL)

**Figure 2:**
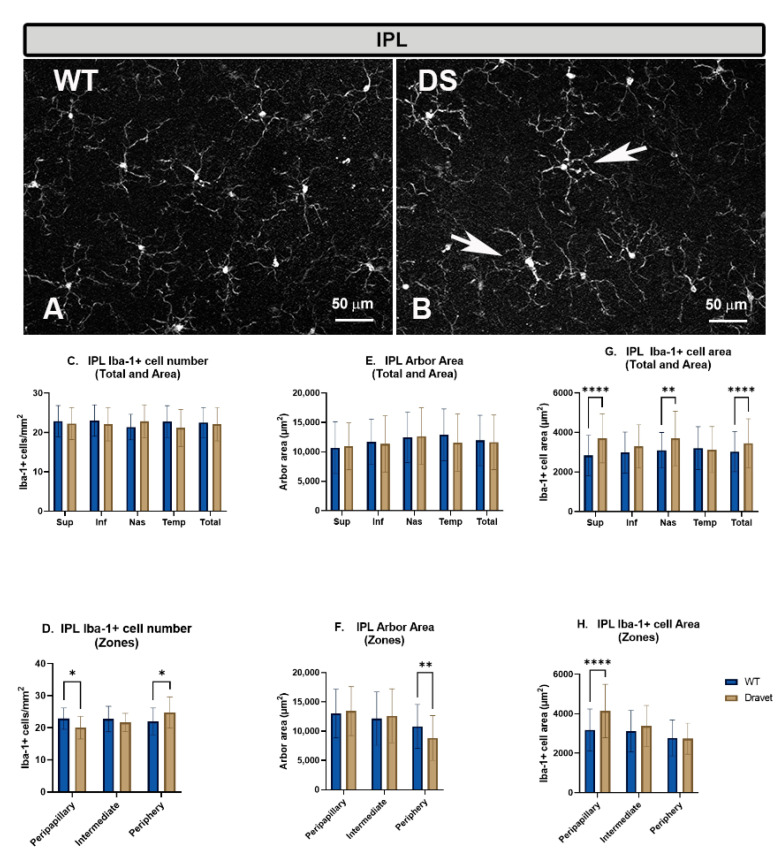
Microglial cells in the inner plexiform layer (IPL). (**A**,**B**): retinal whole-mounts labeled with anti-Iba-1 at 20× magnification. Arrows indicate Iba-1+ microglial cells with robust appearance in the Dravet syndrome (DS) animals. (**C**,**D**): number of Iba-1+ microglial cells per retinal area 0.1502 mm^2^ by total retina and area (**C**) and by zone (**D**). (**E**,**F**): Arbor area of Iba-1+ microglia by total retina and area (**E**) and by zone (**F**). (**G**,**H**): Iba-1+ cell area by total retina and area (**G**) and by zone (**H**). Two-way ANOVA. Data expressed as mean value (±SD). * *p* < 0.05, ** *p* < 0.01, **** *p* < 0.0001. Number of retinas used in the experiment: WT animals n = 6, DS animals n = 6. Sup: superior; Inf: inferior; Nas: nasal; Temp: temporal.

As in the OPL, in the IPL of DS and WT animals, the microglial cells formed a plexus. The microglial cells had a triangular soma from which processes emerged. The processes were divided into primary, secondary, and tertiary, which became finer as they were subdivided (Figure 2A,B).

The **number of microglia** in the IPL showed no significant changes in the DS compared to the WT animals, both in the total number of cells per retina (Figure 2C) and when the analysis was performed by areas (superior, inferior, nasal, and temporal) (Figure 2C). However, the analysis by zones showed a significant decrease (*p* < 0.05) in the number of microglia in the peripapillary area in the DS with respect to the WT animals, while in the periphery, there was a significant increase (*p* < 0.05) (Figure 2D).

The quantification of the **arbor area** in the IPL (indicator of cellular processes retraction) showed no significant changes in the DS compared to the WT animals, both in the total number of cells per retina (Figure 2E) and in the analysis by areas (superior, inferior, nasal, and temporal) (Figure 2E). However, the only zone that showed significant changes in DS with respect to WT was the peripapillary area, in which zone there was a significant decrease (*p* < 0.01) (Figure 2F).

The quantification of the **area occupied by each microglial cell** in the IPL showed a significant increase in the DS compared to the WT animals in the total retina (*p* < 0.0001) (Figure 2G) and in the superior (*p* < 0.0001) and nasal areas (*p* < 0.01) (Figure 2G), as well as in the peripapillary zone (*p* < 0.0001) (Figure 2H). This increase in the area occupied by each microglial cell, indicative of a thickening of the soma and microglial processes, is a morphological sign indicative of microglial activation.

#### 2.1.3. Nerve Fiber Layer-Ganglion Cell Layer (NFL-GCL)

**Figure 3:**
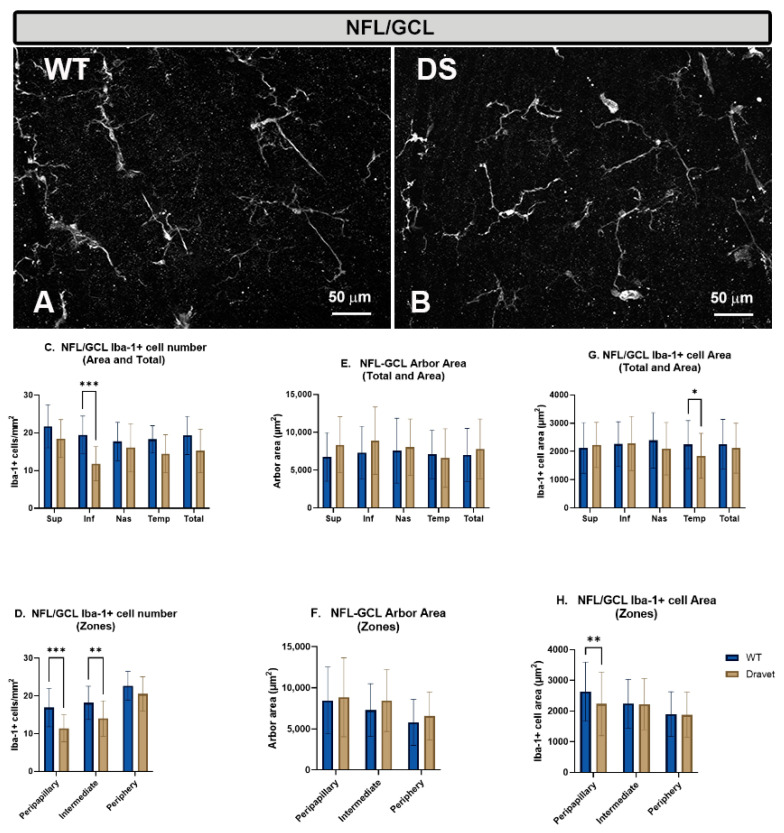
Microglial cells in the nerve fiber layer-ganglion cell layer (NFL-GCL). (**A**,**B**): retinal whole-mounts labeled with anti-Iba-1 at 20× magnification. No major changes were observed between the Dravet syndrome (DS) and the WT animals. (**C**,**D**): number of Iba-1+ microglial cells per retinal area 0.1502 mm^2^ by total retina and area (**C**) and by zone (**D**). (**E**,**F**): Arbor area of Iba-1+ microglia by total retina and area (**E**) and by zone (**F**). (**G**,**H**): Iba-1+ cell area by total retina and area (**G**) and by zone (**H**). Two-way ANOVA. Data expressed as mean value (±SD). * *p* < 0.05, ** *p* < 0.01, *** *p* < 0.001. Number of retinas used in the experiment: WT animals n = 6, DS animals n = 6. Sup: superior; Inf: inferior; Nas: nasal; Temp: temporal.

In the NFL-GCL, the microglial cells of the WT animals are arranged parallel to the retinal surface, but do not form a regular mosaic-like plexus as in OPL and IPL. The microglia of this layer are arranged on the surface of the vessels, which are located in this layer, or in the intervascular areas. In addition, these cells have small somas from which long primary processes emerge, that in turn give rise to secondary processes (Figure 3A).

In this layer in the DS animals, there are apparently no major morphological changes in microglial cells with respect to the WT animals (Figure 3B). 

However, the quantitative study of the **number of microglial cells** in this layer showed there was a significant decrease in the DS animals in the inferior area (*p* < 0.001) (Figure 3C), and in the peripapillary (*p* < 0.001) and intermediate (*p* < 0.01) zones with respect to WT (Figure 3D).

With respect to the **arbor area**, no significant changes were observed in the total retina (no retraction) (Figure 3E), neither in the detailed analysis by areas (Figure 3E) nor by zone (Figure 3F).

The analysis of the **area occupied by each microglial cell** in this layer showed that there was only a significant decrease in this area in the DS animals with respect to the WT in the temporal area (*p* < 0.05) (Figure 3G) and in the peripapillary zone (*p* < 0.05) (Figure 3H), with no changes in the total retina (Figure 3G).

### 2.2. Morphology and Distribution and Quantification of Retinal Astrocytes

In both the WT and DS animals, we observed virtually no GFAP+ labeling in Müller cells, as the terminal feet of these cells were not labeled in the retinal whole-mounts. We also did not find this labeling in the areas where we can see the entire columns of Müller glia, such as the areas where we made a cut to flatten the retinal whole-mount, where the pressure of the slide on the tissue makes it look like a histological section.

In both WT and DS animals, astrocytes were labeled with anti-GFAP.

**Figure 4:**
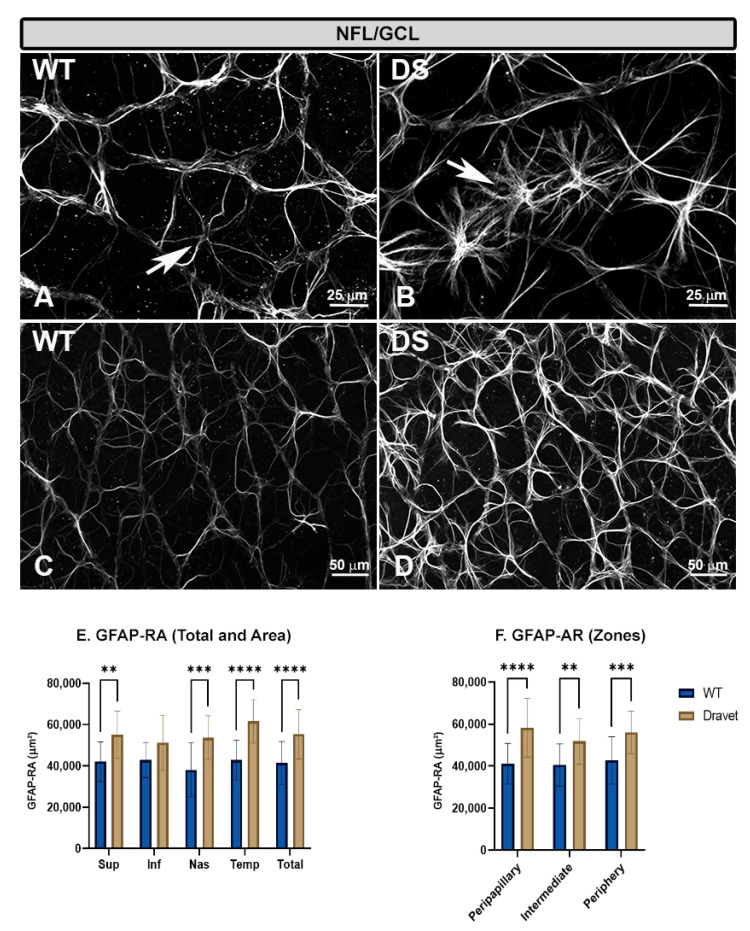
Astrocytes in the nerve fiber layer-ganglion cell layer (NFL-GCL). (**A**–**D**): retinal whole-mounts labeled with anti-GFAP at 20× magnification. (**A**,**C**) WT animals; (**B**,**D**) Dravet syndrome (DS) animals. Arrows indicate GFAP+ astrocytes. In the DS animals, the astrocytes were more robust and had thicker and much more numerous processes than in the WT animals, giving a denser appearance to the astroglial plexus. (**E**,**F**): Area of retina occupied by GFAP (GFAP-RA) by total retina and areas (**E**) and by zones (**F**). Two-way ANOVA. Data expressed as mean value (±SD). ** *p* < 0.01, *** *p* < 0.001, **** *p* < 0.0001. Number of retinas used in the experiment: WT animals n = 6, DS animals n = 6. Sup: superior; Inf: inferior; Nas: nasal; Temp: temporal.

In the WT animals, the astrocytes had rounded cell bodies from which numerous primary and secondary processes emerged radially, giving the cell a stellate appearance (Figure 4A). The astrocytes joined to form a honeycomb-like astroglial plexus extending from the optic disc (peripapillary zone) to the retinal periphery. This plexus was located in the NFL-GCL layer, and in it the cells distinguished themselves from each other and demarcated the course of the vessels by arranging the soma or processes over them (Figure 4C).

In the DS animals, the astrocytes also had a stellate appearance but were more reactive than in the WT animals, as they had more robust somas and the processes were thicker and much more numerous (Figure 4B). This made the astroglial plexus much denser throughout the retina, from the peripapillary zone to the peripheral retina (Figure 4D).

The higher activation of astrocytes in the DS with respect to the WT animals was confirmed as we found a significant increase in GFAP-labelled retinal area (GFAP-RA) when analyzed by total retina (*p* < 0. 0001) (Figure 4E), by retinal areas (superior [*p* < 0.01], nasal [*p* < 0.001] and temporal [*p* < 0.0001]) (Figure 4E), and also by retinal zones (peripapillary [*p* < 0.0001], intermediate [*p* < 0.01] and peripheral [*p* < 0.001]) (Figure 4F).

### 2.3. Quantitative Study of Retinal Ganglion Cells (RGC) and Amacrine GABAergic Cells

**Figure 5:**
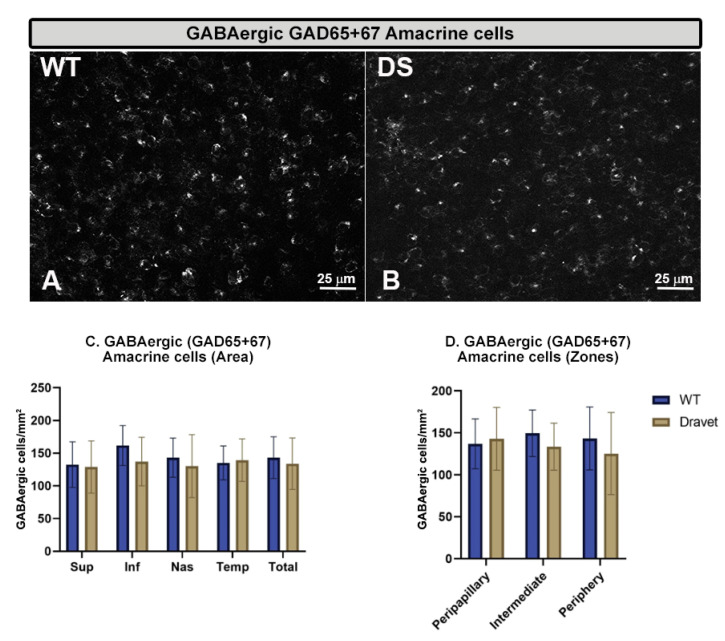
Retinal ganglion cell (RGC) number. (**A**,**B**): retinal whole-mounts labeled with anti-Brn3a at 20× magnification in WT animals (**A**) and in Dravet syndrome (DS) animals (**B**). (**C**,**D**): number of Brn3a + RGCs per retinal area 0.1502 mm^2^ by total retina and areas (**C**) and by zones (**D**). Two-way ANOVA. Data expressed as mean value (±SD). Number of retinas used in the experiment: WT animals n = 6, DS animals n = 6. Sup: superior; Inf: inferior; Nas: nasal; Temp: temporal.

**Figure 6:**
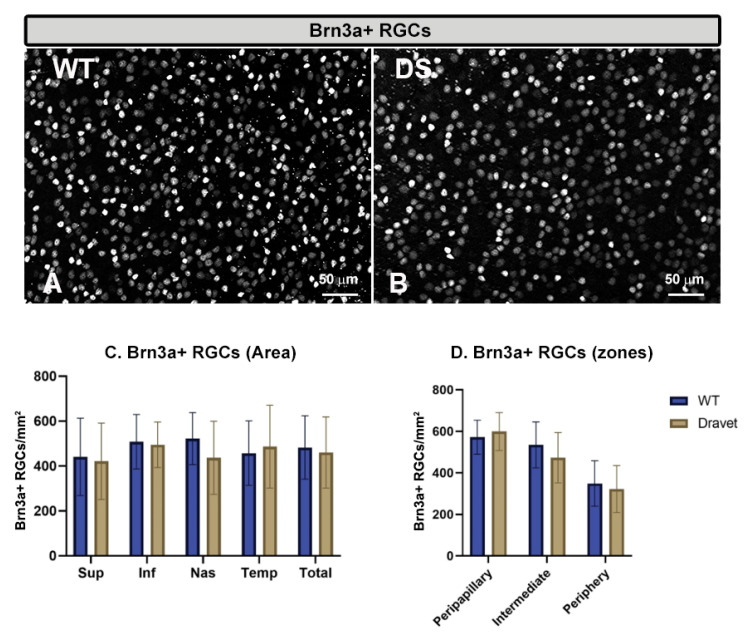
Retinal GABAergic amacrine cell number. (**A**,**B**): retinal whole-mounts labeled with anti-GAD65+67 at 40× magnification in WT animals (**A**) and in Dravet syndrome (DS) animals (**B**). (**C**,**D**): number of GABAergic GAD65+67 amacrine cells per retinal area 0.0376 mm^2^ by total retina and areas (**C**) and by zones (**D**). Two-way ANOVA. Data expressed as mean value (±SD). Number of retinas used in the experiment: WT animals n = 6, DS animals n = 6. Sup: superior; Inf: inferior; Nas: nasal; Temp: temporal.

The quantitative study of the **number of Brn3a+ RGCs** (Figure 5) and the **number of GABAergic GAD65+67+** amacrine cells (Figure 6) showed that in both cell types there were no significant changes in the DS with respect to the WT animals (*p* > 0.05).

## 3. Discussion

Dravet syndrome is a very severe early-onset epileptic encephalopathy resistant to antiepileptic drug treatment. Seizures begin a few months after birth and in 80% of patients are caused by a mutation in the *Scn1a* gene, encoding the alpha subunit of the voltage-gated sodium channel Nav1.1. GABAergic interneurons of the central nervous system are predominantly affected by the mutation, causing cerebral hyperexcitability and seizures, leading to cognitive impairment [10].

Numerous murine models have been developed given the genetic origin of this disease [12,13,39,40]. One of them is a heterozygous conditional knock-in model carrying a missense mutation (A1783V) in the *Scn1a* gene expressed exclusively in neurons of the Central Nervous System (Syn-Cre/Scn1a^WT/A1783V^) [22]. In this model, spontaneous epileptic activity is evident and motor and cognitive impairment are manifested as long-term comorbidities. A large body of preclinical and clinical evidence relate neuroinflammation to drug-resistant epilepsy [41,42,43], which is also observed in these animals.

This is a preliminary study that describes for the first time that astroglial and microglial activation occur in the retina of Syn-Cre/Scn1a^WT/A1783V^ mice with DS, in order to establish a correlation between cell alteration in the retina and that seen in CNS.

The retina has a complex structure with different cell populations and several neuronal layers interconnected by synapses. Astrocytes and Müller glia support the retinal cells and perform important activities for the perfect functioning of the retina. These two cell populations are essential for information processing in neuronal circuits as they maintain homeostasis of extracellular ions and neurotransmitters such as glutamate and GABA, participate in glucose metabolism, remove metabolic waste products in the retina, regulate local blood flow, and induce the blood-retinal barrier. In addition, they play a key role in the local immune response and protect neurons from oxidative damage [44]. From a structural point of view, astrocytes are mainly located in the NFL-GCL of the retina [45,46].

Somas of Müller glia in the inner nuclear layer (INL) of the retina are arranged radially occupying practically the entire retinal thickness, from the outer limiting membrane (OLM), where their apical ends are located, to the inner limiting membrane (INL), where their basal ends terminate [47,48,49]. The lateral processes of Müller glia expand into the inner and outer plexiform layers (IPL and OPL, respectively), where they form sheaths around synapses in these layers.

Astrocytes are the most abundant glial cells in the brain and are activated in epilepsy processes. The release of proinflammatory cytokines by reactive astrocytes may contribute to increased cellular excitability and therefore may play an important, possibly central, role in epileptogenesis. Indeed, astrocyte activation is present in a drug-resistant epilepsy such as temporal lobe epilepsy [50], and also in different animal models of epilepsy. In this study we have evidenced the existence of increased astroglial activity in the nerve fiber layer-ganglion cell layer (NFL-GCL) of the retina, which correlates positively with that found in the brain of these same animals [22]. The area of the retina occupied by astrocytes is larger in DS animals than in healthy animals, with a much denser astroglial plexus extending from the peripapillary area to the peripheral retina in the different retinal sectors (superior, inferior, nasal and temporal). In addition, the astrocytes show more robust soma with thicker and more numerous processes, thus confirming a higher astrocyte activation also in the visual system of the animals.

In our DS model, it appears that there is no severe retinal damage associated with Dravet’s pathology, since Müller glia do not express GFAP which is a sign of retinal damage, but we cannot state that Müller glia do not undergo other types of alterations in DS. However, signs of microglial activation were found in the retina as there was a significant decrease in the microglial arbor area (indicating a retraction of the microglial processes) in all areas (superior, inferior, nasal and temporal) and zones (peripapillary, intermediate and peripheral) in the outer plexiform layer (OPL) of the retina. In addition, there was also a significant increase in the area occupied by each microglial cell in the outer plexiform layer (OPL) in the total retina, in the superior and inferior areas and in the peripapillary zone, indicating increased soma thickness. This microglial activation only seems to affect the plexiform layers since the analysis of the nerve fiber layer-ganglion cell layer (NFL-GCL) of the DS animals showed that the number of microglial cells was even lower in the inferior area and in the peripapillary and intermediate areas of the retina without altering their arrangement along the vessels. This localized microglial activation was also observed in the brain of these animals and was restricted to the prefrontal cortex and dentate gyrus of the hippocampus being absent in the different subfields (CA1, CA2 and CA3) of the hippocampal Amon’s horn and in the striatum [22]. This may indicate the existence of a close relationship between the prefrontal cortex, the hippocampal dentate gyrus and the retina in our preclinical model of SD and could be supported by a recent study conducted in subjects at high risk of developing Alzheimer’s disease, in which changes at the retinal level have been found to correlate with alterations in affected brain areas when mild cognitive impairment is present [51]. These findings show the retina as the “window” through which DS patients’ brain can be visualized.

Although in our DS model microglial activation is evident [1,40], neuronal loss is not a prominent feature in DS [52]. The fact that both types of retinal neurons (RGCs and GABAergic amacrine cells) do not die does not mean they function properly in DS animals. In these animals, as in 80% of DS patients, a mutation occurs in the *Scn1a* gene, which encodes the α-subunit of the voltage-dependent sodium channel Nav1.1 [3,10]. This loss of function in Nav1.1 results in decreased sodium current and altered activation of many types of GABAergic interneurons [10,11], leading to an imbalance between excitation and inhibition, contributing to hyperexcitability and seizures [9]. In our experimental model, the *Scn1a* gene mutation is not exclusive to GABAergic neurons but can be expressed in different neurons of the CNS [22]. Nav sodium channels, including the Nav1.1 sodium channel, are also expressed in the retina [53,54]. These channels have been observed in goldfish retinal bipolar neurons and may play a role in synaptic signaling [55]. It has been observed in humans that mutations in the *Scn1a* gene, which alter neuronal excitability in the brain, can occasionally alter the excitability of retinal cells [56]. An alteration of retinal bipolar cells, which are located in the inner nuclear layer of the retina and perform their synapses at the OPL and IPL level, could cause the microglial changes observed at the OPL and IPL level. In addition, an alteration of these channels in GABAergic amacrine cells, whose synapses are located in the IPL, could also be contributing to this microglial activation. However, to corroborate all this, studies on the alteration of these channels in the different retinal neurons in DS models would have to be performed.

Photosensitivity is present in 40% of patients with DS [57] and changes in visual function have been described before the onset of cognitive impairment and even before the clinical confirmation of this epileptic syndrome [37,38]. At the present time, minimally invasive approaches such as optical coherence tomography (OCT) allow the detection of morphological changes in the retina [32,35,51,58]. If the relationship between brain and retinal involvement in DS is corroborated, the evolution of the disease could be monitored using the OCT technique and retinal cells could become a good biomarker of prognosis and predictors of treatment response.

To summarize, this work describes for the first time histological changes in the retina associated with the pathology of Dravet syndrome and is the preliminary step for subsequent studies to evaluate: (i) the impairment of retinal neurons in DS; (ii) the inflammatory or anti-inflammatory function of the reactive retinal microglia; and (iii) retinal changes in animal models of DS and patients measured by the OCT technique useful to carry out the follow-up of DS patients using a minimally invasive technique.

## 4. Materials and Methods

### 4.1. Animals and Genotyping

In this study, we used conditional knock-in mutant mice (knock-in mutation A1783V in Nav1.1 protein) generated by Cre-LoxP technology, in which the *Scn1a* gene is primarily mutated in neuronal cells. For this purpose, B6(Cg)-Scn1atm1.1Dsf/J mice (heterozygous for the transgene, JAX stock #026133) were crossed with Cre-recombinase linked to synapsin-1 promoter mice (CreB6.Cg-Tg (Syn1-cre)671Jxm/J; JAX stock #003966), both in C57BL6/J background and acquired from The Jackson Laboratory (Bar Harbor, ME, USA).

Two experimental groups were established from the offspring obtained in the crossings: Syn-Cre/Scn1aWT/A1783V mice, which carry the A1783V mutation in the Nav1.1 protein exclusively in neurons showing the pathological phenotype; and Scn1aWT/WT mice (wild type not expressing Cre) as the unique control group [22].

Approximately 15 litters were needed to generate the number of animals required for the experiments and, in both cases, all animals born in each litter were proportionally distributed for the two genotypes.

In addition, our experimental groups for this study were composed with equivalent proportions of male and female mice in all cases since, as in patients [2], the incidence and severity of this disease does not respond to gender in our murine model [22].

Genotypes were verified by polymerase chain reaction (PCR) using genomic DNA from mouse tail biopsies. Genomic DNA was extracted and amplified using REDExtract-N-Amp Tissue PCR kit (Sigma-Aldrich, Madrid, Spain), following manufacturer’s instructions and as published elsewhere [22,59].

During the experiments, mice were housed in a climate-controlled room (21 ± 2 °C and 60% humidity) under a controlled photoperiod of 12 h light/12 h dark (08:00–20:00 light). All animals had ad libitum access to standard chow and water. All experiments were conducted according to national and European guidelines (RD 53/2013 and directive 2010/63/EU, respectively), followed the principles of the ARRIVE and 3R guidelines, and were approved by the Animal Welfare Committee of the Complutense University and the “Comunidad de Madrid” (ref. PROEX 033/17). In addition, animal procedures followed institutional guidelines, European Union regulations for the use of animals in research, and the Association for Research in Vision and Ophthalmology (ARVO) statement for the use of animals in ophthalmic and vision research. All possible efforts were made to minimize animal pain and discomfort, as well as reduce the number of experimental subjects.

Animals were divided into the following groups: Scn1aWT/WT mice (WT) as control animals (*n* = 6) and Syn-Cre/Scn1a^WT/A1783V^ (DS) mice as Dravet syndrome animals (*n* = 6).

### 4.2. Immunohistochemistry

The animals were anesthetized with an intraperitoneal injection of ketamine (75 mg/kg; Anesketin^®^, Dechra Veterinary Products SLU, Barcelona, Spain) and medetomidine (0.26 mg/kg; Medetor^®^, Virbac España S.A., Barcelona, Spain) and then perfused transcardially through the ascending aorta first with saline and then with 4% paraformaldehyde in 0.1 M phosphate buffer (PB, pH 7.4).

Once the animals were perfused and before removal of the eyeballs, the upper eyelid was sutured to maintain the orientation of the eyes. The nasal caruncle and extraocular rectus muscles were also used as additional landmarks for eye orientation. Once the eyes were removed, they were postfixed for 24 h in the same fixative and then transferred to 0.1 M PBS solution at 4 °C. Subsequently, the cornea and lens were removed from the eyeball and retinas were extracted from the resulting optic cup for performing retinal whole-mounts. The retinas were then frozen but were previously cryoprotected by introduction into sucrose at increasing concentrations (10%, 20%, and 30%) for 1 h, 2 h, and overnight, respectively. Freezing was performed using liquid nitrogen and then the tissue was stored at −80 °C until use.

To analyze the different morphological signs of microglial activation, we used the antibody against ionized calcium binding adaptor molecule 1 (Iba-1), which allows morphological study of microglia [60]. To study the activation of astroglial cells, we used the antibody against gliofibrillary acidic protein (GFAP), which is the main constituent of the intermediate filaments of astrocytes [61]. To quantify the number of RGCs, we used an antibody against the brain-specific homeobox/POU domain protein 3A (Brn3a), a marker that localizes to the nuclei of RGCs and whose expression decreases when a cell dies [62]. To quantify the number of GABAergic amacrine cells, we used an antibody against GAD65/67. GAD is highly expressed in GABAergic neurons, and exists as two isoforms, GAD65 and GAD67 (molecular masses of 65 and 67 kDa, respectively) that are encoded by two distinct genes [63].

Retinal whole-mounts were immunostained according to previously-used protocols [64]. In summary, left retinas were double-immunostained with rabbit polyclonal anti-Iba-1 at 1:600 dilution (ref. 01919741, Wako, Osaka, Japan) and mouse monoclonal anti- GFAP clone GA5 at 1:150 dilution (ref. MAB3402 Millipore, Massachusetts, MA, USA), while the right retinas were double-immunostained with mouse anti-Brn-3a at 1:300 dilution (ref. MAB1585, Sigma-Aldrich, Darmstadt, Germany) and rabbit polyclonal anti-GAD65+GAD67 at 1:50 dilution (ref. AB183999, ABCAM, Cambridge, MA), followed by the corresponding secondary antibodies: donkey anti-rabbit IgG Alexa Fluor 594 (1:800; ref. A21207; Invitrogen, Paisley, UK), goat anti-mouse Alexa Fluor 488 (1:200; ref. A11001; Invitrogen, Paisley, UK), goat anti-mouse IgG1 Alexa Fluor 488 (1:150; ref. A21125, Invitrogen, Carlsbad, CA, USA) and donkey anti-rabbit IgG Alexa Fluor 594 (1:150; ref: A21207; Invitrogen, Carlsbad, CA, USA). To dilute the primary antibodies, a solution containing 1% of the serum of the animal where the secondary antibody was developed along with triton-x 100 and PBS was used. The secondary antibodies were diluted in PBS. 

Negative controls were made to see that the primary antibody reacted only with its primary antibody. In the first, the primary antibody was not added, and the tissue was incubated with the primary antibody diluent and then with the secondary antibody. In the second, the secondary antibody was omitted, and the tissue was incubated only with the primary antibody and then with the secondary antibody diluent. We also made a third control to evaluate the amount of endogenous fluorescence of the tissue, and for this purpose neither the primary nor the secondary antibodies were added, and the retinas were incubated in the corresponding diluent solutions of the antibodies.

An ApoTome device (Carl Zeiss, Munich, Germany) and a high-resolution digital camera (Cool-SNAP Photometrics Tucson, AZ, USA) coupled to a fluorescence microscope (Axioplan 2 Imaging Microscope Carl Zeiss, Munich, Germany), as previously described [65], were used to study and photograph the retinas. The microscope was equipped with the appropriate filters for the different emission spectra, Alexa Fluor 488 (Filter set 10, Zeiss) and Alexa Fluor 594 (Filter set 64, Zeiss). The ApoTome device makes it possible to obtain high-quality photographs of thick tissues. In thick tissues, obtaining quality images can be more difficult due to the fluorescence signal occurring outside the focal plane, resulting in reduced contrast and resolution of the axial dimension (*z*-axis). The ApoTome allows imaging as if it was an optical section, which improves the contrast and resolution of the images. The ApoTome projects a grating in the focal plane of the objective that moves to three different positions on the sample, thus relying on the theory of interferometry. The microscope’s ZEN2 software (Carl Zeiss AG, Oberkochen, Germany) processes the images by removing everything that is out of focus. With this method we obtain a better-quality image that is similar to an optical section in the plane of focus.

#### 4.2.1. Retinal Quantifications

**Figure 7:**
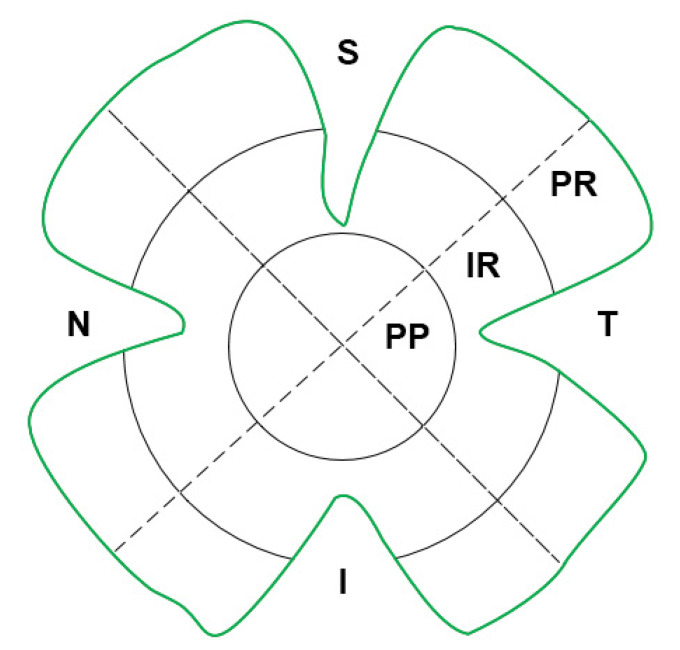
Schematic representation of the areas: superior (S), inferior (I), nasal (N) and temporal (T); and zones: peripapillary (PP), intermediate (IR) and peripheral (PR), into which the retina has been divided for study.

Three different metrics were used for all quantifications: (i) total retinal area, (ii) analysis by retinal areas (superior, inferior, nasal and temporal). and (iii) analysis by retinal zones (peripapillary, intermediate and peripheral) (Figure 7).

#### 4.2.2. Microglial Quantifications

When a microglial cell is activated, it undergoes a series of morphological changes. The cells increase the size of the soma and its processes, indicating a greater cellular activity. They also retract their processes, in order to move more easily, reaching in cases of maximum activation to acquire an amoeboid morphology. In addition, it also proliferates, thus increasing its numbers in the tissue. In retinal tissue, we quantified morphological signs of microglial activation in the outer plexiform layer (OPL), in the inner plexiform layer (IPL), and in the nerve fiber layer-ganglion cell layer (NFL-GCL), which are the retinal layers where microglial cells form cellular plexuses. These signs included: (i) the number of microglial cells; (ii) the area occupied by each microglial cell (that would indicate whether there are variations in soma size and processes); and (iii) the arbor area of microglial cells, to determine whether there is retraction of the microglial cell processes. These quantifications were made in the 6 animals of the WT group and in the 6 animals of the DS group.

Most of the microglial cells were arranged parallel to the retinal surface, which facilitated their complete visibility in the retinal whole-mounts. We placed the retinas with the vitreous side up, so that when we started focusing the retinal whole-mount, the first microglial plexus would be the NFL-GCL plexus, followed by the IPL plexus, and finally the OPL plexus. With this system it is easy to differentiate the different microglial plexuses in retinal whole-mounts. In addition, the microglial cells differ somewhat in their morphological features in each retinal layer where they are located, which also helped us to differentiate the retinal layer we were analyzing.

Using the motorized stage and camera associated with the microscope, a series of fields in the retinal whole-mount were photographed at 20x, giving each field an area of 0.1502 mm^2^. Three equivalent fields were photographed for each horizontal and vertical meridian (crossing the optic nerve). These fields included the superior, inferior, nasal and temporal areas of the retina along the x-y axis, so a total of 12 sectors per retina were analyzed (3 zones × 4 areas = 12 sectors). Since three plexuses (OPL, IPL and NFL-GCL) were analyzed per retina, a total of 36 (12 × 3 = 36) microphotographs were obtained per retina. By studying 6 retinas for each experimental group (36 × 6), 216 microphotographs were obtained for each experimental group. To obtain an image with a greater depth of focus that would add up the different images obtained in the *z*-axis with good focus, we used the Extended Focus module of the ZEN2 software (Carl Zeiss AG, Oberkochen, Germany).

##### Number of Microglia Iba-1+

An increase in the number of microglial cells is a sign of microglial activation. For this quantification, in all images obtained at 20× of the different layers of the retina analyzed (OPL, IPL and NFL-GCL) of the WT and DS groups, Iba-1+ cells were manually counted using the interactive manual counting tool “Interactive Measurement”, included in the AxioVision Release 4.8.2 software (Zeiss, Munich, Germany) associate with the fluorescence microscope. The value obtained was the number of cells per area (0.1502 mm^2^).

##### Area Occupied by Each Microglial Cell

The area occupied by each microglial cell indicates whether a microglial cell increases the thickness of the soma and its processes. If the soma and processes are thicker, they cover a larger area. For the measurement of the area of each microglial cell, we used the protocol of [36]. For this, we used the ImageJ program (v. 1.52u 2020) (National Institutes of Healt, Bethesda, MD, USA), which is a Java program for image processing. Three randomly-selected whole microglial cells were measured in each of the images obtained at 20x for all layers analyzed (OPL, IPL, NFL-GCL). The only criterion we used was that the cell had to be complete, i.e., the entire soma and processes had to be within the field of analysis.

The protocol we followed was as follows. First, we changed the images to grayscale to obtain better visibility of the positive staining; then, we adjusted the brightness/contrast to obtain images in which all microglial cells and their processes could be correctly visualized. 

We used the thresholding tool to convert the images to binary, thus making the positive staining more evident, and used the “wand tool” to select each microglial cell for which we wanted to measure its area. Subsequently, the area of positive staining was quantified using the ROI manager, thus obtaining the total area occupied by each microglial cell.

##### Arbor Area

We use the arbor area of the microglial cells to analyze whether there is an elongation or retraction of the microglial processes, regardless of whether the cell is thicker or thinner. If there is a retraction of the processes the arbor area becomes smaller than if the processes are long.

For these measurements we also followed the protocol of [36]. The same fields of measurements and the same processing methods were used as for “area occupied by each microglial cell”. Three randomly-selected whole microglial cells were also quantified for the different layers analyzed (OPL, IPL, NFL-GCL). The selection criterion used was that the cell had to be complete, i.e., the entire soma and processes had to be within the field of analysis. In each selected cell, we used the “polygon tool” to delimit the outline of the microglia with a polygon connecting the most distal cell processes. The delimited areas were measured with the “ROI manager”, thus obtaining the total area of the arbor area of each microglial cell.

### 4.3. Astroglial Quantifications

To analyze whether astroglial cell activation occurred, we measured the GFAP-labeled retinal area (GFAP-RA) using a previous protocol [64].

To obtain the images we used to measure GFAP-RA we proceeded in the same way as for the microglial cells. The retinal whole-mounts were photographed at 20× giving each field an area of 0.1502 mm^2^. Three equivalent areas were photographed in each horizontal and vertical meridian, including the superior, inferior, nasal and temporal areas of the retina along the x-y axis. Therefore, 12 sectors were photographed per retina (4 areas × 3 zones = 12 sectors) but since we studied 6 animals per group, a total of 72 images were obtained for analysis.

To quantify the GFAP-RA we used a thresholding tool in MATLAB on each image. The thresholds determine the pixels of the objects of interest based on the grayscale values, allowing us to differentiate them from other areas of the image based on the grayscale values of the images. Subsequently, we quantified the GFAP-RA using an algorithm developed by our group in the MATLAB environment [66].

### 4.4. Brn3a+ RGC and GABAergic GAD65/67 Amacrine Cells Quantifications

To perform the measurements, we used the Fernández-Albarral protocol [67]. To quantify the number of RGCs or the number of GABAergic GAD65/67 amacrine cells in each retinal whole-mount in the two experimental groups (WT and DS), photographs at 20× for the study of RGCs and at 40× for the study of GABAergic amacrine cells were taken. Three images were taken for each area (superior, inferior, nasal and temporal), which gave us a total of 12 images per retina. As six retinas were analyzed per experimental group, a total of 72 microphotographs were obtained for each antibody used. Each image at 20× provided an area of 0.1502 mm^2^ per field, and each image capture at 40× corresponded to an area of 0.0376 mm^2^.

For the quantification of Brn3a+ RGCs and GABAergic amacrine cells GAD65/67, we applied the same algorithm developed in MATLAB for an automatic microglial cell counting [66]. In this case, we specified the minimum distance between RGCs or GABAergic amacrine cells so that the program distinguishes them as one cell and counts them only once. Briefly the counting protocol was as follows. The selected images were averaged as a z-stack, resulting in a z-projection, which was processed in two ways to preserve the cell body labeling. In the first step, the image was normalized to the pixel with the maximum image value, so that the image values fell within a range from 0 to 1. Next, a threshold was applied and all values <0.2 were set to 0, while the remaining values were preserved. The remaining image was segmented and the center of mass of each segment was determined to identify the presence or absence of cells. To avoid multiple counting of the same cell in adjacent segments, the minimum distance at which cells were arranged from each other was specified. All points closer to each other than this minimum distance were considered to belong to the same cell and were counted only once.

### 4.5. Statistical Analysis

The assessment of normal distribution of data was carried out with the Shapiro–Wilk test. Levene’s test was used to assess the homogeneity of variance between groups. Statistical significance between groups was assessed depending on the distribution by two-way ANOVA with the Tukey post hoc test, for multiple comparisons. ANOVA post hoc tests were carried out only if F had a *p* < 0.05, and no significant variance in homogeneity was found within analyzed groups. Differences were considered statistically significant for *p*-values < 0.05. Statistical analysis was carried out with GraphPad Prism v.9 (GraphPad Software, La Jolla, CA, USA), and the same software was used to design graphs. The notations used for the different levels of significance were * *p* < 0.05, ** *p* < 0.01, *** *p* < 0.001, **** *p* < 0.0001.

## 5. Conclusions

We can conclude that in the DS model (Syn-Cre/Scn1a^WT/A1783V^) changes occur in the retina, such as activation of astroglial and microglial cells, but not death of RGCs and GABAergic amacrine cells. These changes are similar to those found at the level of the prefrontal cortex and the dentate gyrus of the hippocampus in the same experimental model in PND25, which could indicate a relationship between brain and retinal changes in DS. This opens the possibility that in the future, if it is demonstrated that the changes that are occurring in the retina could be detected with non-invasive techniques such as OCT, the images obtained could serve as a possible biomarker for treatment response in DS.

## Data Availability

The data supporting the findings of this study are available from the corresponding author upon request.
